# Novel microsatellite markers for *Distylium lepidotum* (Hamamelidaceae) endemic to the Ogasawara Islands

**DOI:** 10.1186/s13104-016-2137-9

**Published:** 2016-07-02

**Authors:** Kyoko Sugai, Suzuki Setsuko

**Affiliations:** Laboratory of Wildlife Ecology, Forestry and Forest Products Research Institute, 1 Matsunosato, Tsukuba, Ibaraki 305-8687 Japan; Laboratory of Ecological Genetics, Forestry and Forest Products Research Institute, 1 Matsunosato, Tsukuba, Ibaraki 305-8687 Japan

**Keywords:** *Distylium lepidotum*, Next-generation sequencing, Ogasawara Islands, Population genetics, Simple sequence repeat

## Abstract

**Background:**

*Distylium lepidotum* is a small tree endemic to the Ogasawara Islands located in the northwestern Pacific Ocean. This species is a sole food for an endemic locust, *Boninoxya anijimensis*. Here, we developed microsatellite markers to investigate genetic diversity and genetic structure and to avoid a genetic disturbance after transplantation to restore the Ogasawara Islands ecosystem.

**Results:**

Microsatellite markers with perfect dinucleotide repeats were developed using the next-generation sequencing Illumina MiSeq Desktop Sequencer. Thirty-two primer pairs were characterized in two *D. lepidotum* populations on Chichijima and Hahajima Islands of the Ogasawara Islands. The number of alleles for the markers ranged from three to 23 per locus in the two populations. Expected heterozygosity per locus in each population ranged from 0.156 to 0.940 and 0.368 to 0.845, respectively.

**Conclusions:**

These microsatellite markers will be useful for future population genetics studies of *D. lepidotum* and provide a basis for conservation management of the Ogasawara Islands.

## Findings

### Background

Microsatellite markers, or simple sequence repeats, are widely applicable as DNA-based markers for population genetics studies. Moreover, their cost-effective development has been increasingly facilitated by applying next-generation sequencing (NGS) technologies [20].

*Distylium lepidotum* Nakai (Hamamelidaceae) is a small tree endemic to the oceanic Ogasawara Islands in the northwestern Pacific Ocean. The species is the dominant tree in the *Distylium*–*Pouteria* dry scrub [18], which is inhabited by *Boninoxya anijimensis* Ishikawa, a locust recorded as a new genus and species [8]. The locust utilizes *D. lepidotum* as the sole food, i.e., it is monophagous [8, 9]. Although it is only distributed on Anijima Island of the Ogasawara Islands, it has been exposed to alien predatory species such as *Anolis carolinensis*. Conservation/benign introduction measures of *B. anijimensis* are needed on the Ogasawara Islands, except Anijima Island, to protect the *B. anijimensis* populations. As *D. lepidotum* is an essential food source, it may be possible to transplant the species. Therefore, it is important to reveal the genetic structure of the species to minimize any genetic disturbance due to the transplant. Here, we developed microsatellite markers to investigate the genetic diversity and structure in *D. lepidotum*.

### Methods

Microsatellite markers were developed for *D. lepidotum* using an Illumina MiSeq Desktop Sequencer (Illumina, San Diego, CA, USA). Total genomic DNA was extracted from one silica-gel dried *D. lepidotum* leaf sample collected from Chibusayama (26°39′17.4″N 142°10′03.6″E) on Hahajima Island of the Ogasawara Islands using a DNeasy Plant Mini Kit (QIAGEN, Hilden, Germany). A shotgun library was prepared using the Nextera DNA Sample Preparation Kit v2 (Illumina), and the raw de novo sequencing data were obtained using the MiSeq Reagent Kit v2 (500 cycles) (Illumina). The raw reads were divided into each index, extra sequences (adapters and indices) were trimmed, and FASTAQ files were generated using the MiSeq Reporter v.2.5.1 (Illumina). The paired-end reads were merged using PEAR 0.9.6 [21] with default parameter settings. After the paired-end assembly, the low quality reads (<95 % with Phred quality score of 30) were removed using the script fastq_quality_filter included in the FASTX-Toolkit v.0.0.14 [7]. The resulting FASTQ files were converted to FASTA format using the ShortRead package [12]. A total of 1734,031 contigs with an average length of 241 bp were obtained.

The microsatellites were identified and the primer pairs were designed with QDD2.1 [11]. A total of 41,367 unique sequences containing pure/compound microsatellite regions (2–6 nucleotide motifs with >5 repeats) and primer-designable flanking regions were selected. The primer pairs were designed with Primer3 [17] and implemented in QDD2.1 using the following criteria: (1) polymerase chain reaction (PCR) product size of 90–500 bp and (2) primer lengths of 20–27 bp, melting temperature of 57–63° C, and GC content of 20–80 %. Finally, 18,239 microsatellite primer pairs were designed using Primer3.

Amplification and polymorphism were confirmed in 48 selected primer pairs after considering the microsatellites (one single dinucleotide motif with more than ten repetitions), design type (“A” or “B” in QDD2.1), and PCR product size to apply multiplex amplification (Table 1). Four universal primers with different fluorescent tags designed by Blacket et al. [1] were prepared, and the 5′ end of each forward primer was attached to the same sequence as a tail. In addition, as the 5′ end sequences of each reverse primer became 5′-GTTT-3′, a PIG-tail (5′-GTTT-3′, 5′-GTT-3′, 5′-GT-3′, or 5′-G-3′) was added to reduce stuttering due to inconsistent addition of adenine by Taq DNA polymerase [2].

**Table 1:** Characteristics of the 32 microsatellite markers developed for *Distylium lepidotum*

Locus	Repeat motif	Forward primer sequence (5′–3′)^a^	Reverse primer sequence (5′–3′)^b^	*T*a (°C)	Size range (bp)	GeneBank accession no.
Isu00524	(CT)30	[tail C] TTTATGCTTATTCACCCTTGAACC	gtttAAACACCCATTAGTTCTTCTGTCTG	57	136–194	LC085250
Isu01062	(TC)25	[tail B] TACGAATGATGGGTCAAACTGTAA	gtttGCCTTAAATTGACTGGAAGTGATT	57	228–270	LC085251
Isu01853	(AG)19	[tail D] CACTAGTTATTGAGGTAGGCGGGT	gTTTGTTAACGAATGAGTTGGGATT	57	274–302	LC085252
Isu03838	(TC)24	[tail D] TTCCTGAAACGGTTACACAATACA	gtttAGTGGAGATGATAAACGGATTGAC	57	111–135	LC085253
Isu04069	(GA)24	[tail B] TTAGATTTGAAGGCGATAAAGGTT	gttTCCTTGATCTGTCCAATGTCA	57	135–171	LC085254
Isu04385	(TC)22	[tail A] AATGGGTCAGTGAGAATCTGTCTT	gtttCAAGGAAATCGTATATGCAGAACA	57	215–245	LC085255
Isu04423	(GA)22	[tail B] AAGCAGAGCTTACCATGATTCACT	gttTAGATCTCTGAGGAGGGACACATT	57	260–308	LC085256
Isu04472	(AG)26	[tail D] ATTTGGATCATCACTCGAGGTAAA	gtTTATTCGTTTGCACTCTTATTTGA	57	214–266	LC085257
Isu04870	(CT)16	[tail B] TTAATTGGTTTCCCATTTGATCTC	gtttCATGCAGATGCAGACTCTAAGAAG	57	285–299	LC085258
Isu04950	(GA)22	[tail A] AGACAATTCTGTGCTCCAGTATCA	gtttAACATTGAAAGTTGAAGACCCAAC	57	263–299	LC085259
Isu04954	(TC)31	[tail A] CTAATCCAAATCAACCCATCTACG	gtttCACCTCTCGTTTACTTCCATTGAT	57	128–156	LC085260
Isu05730	(AT)11	[tail A] ACATCGTCACCTCTATTAACCGAC	gtttCAAGAGATTTCGAAGTGAAACAGA	57	346–366	LC085261
Isu06843	(AG)27	[tail B] GTTGACATCCCTACTCCTCCTACC	gttTCTAAGCAAATGTGCATCGTTAGT	57	96–132	LC085262
Isu07049	(CT)26	[tail A] TCCATGTATTTATTTCGATCCTCC	gtttGGGAAATACCATAAACATAAAGATGG	57	90–134	LC085263
Isu07063	(GA)24	[tail C] AGCTTGCATGAGGTTTCACTAAGA	gtttCGACAACAGTACTAATCAACACGG	57	109–143	LC085264
Isu09807	(GA)23	[tail D] AACGCAAGATTTATCATTACCAGC	gtttAAGACTCTCAAGATCTGTGCCAA	57	213–239	LC085265
Isu09853	(GA)22	[tail D] CAATTCCCTCAATTGTTGTTTCTT	gtttAGAAACTTAAAGACAAACCGGGAT	57	304–326	LC085266
Isu10193	(GA)24	[tail B] ATTTATGTGGAAGTAGTAGCCGGA	gttTACTGCTGGCTTGACATAGAAAGA	57	214–236	LC085267
Isu11459	(AG)19	[tail D] TAAAGCATCAAACAAGCGAATATG	gtttACAATAAGAAAGCGACATGCTCA	57	265–291	LC085268
Isu12115	(GA)11	[tail A] TACGATTCAAGCTTGTCATACTCG	gtttATATTTACGCGCAAACTCTCGC	57	413–417	LC085269
Isu12238	(CT)24	[tail D] CCAAGATTATGCAACCTAAGGAAG	gtttACCCTGAATTCCATCTAGACCTTT	57	116–156	LC085270
Isu12265	(TC)21	[tail C] TGATAGATACATGTCCCACTGTCTT	gttTAAACCTAGCCAAACAAATCCAAC	57	85–121	LC085271
Isu12586	(AT)11	[tail C] TAGACAACTTTCTGGATCAAAGCC	gtttGGCTGTGTATATGTATGCGTGTTT	57	319–359	LC085272
Isu13849	(CT)12	[tail D] CAAGATCAAGATTGAAATGGAATTG	gtttATCCGATAGATCAGTACTTGGTGG	57	326–350	LC085273
Isu13965	(AG)25	[tail B] GTGTAAGTTGTGGGTTTAACGGAT	gtttAAGACATCAGCAAACTAGTCCACC	57	155–183	LC085274
Isu15054	(TC)24	[tail A] CGGGATGTAAACATAGATGTCAAA	gttTATGGCCTAGGAAGATAATGTTGG	57	219–273	LC085275
Isu16246	(CT)26	[tail C] AATCATGTAGCGAGCTTGAACTTT	gtttCATGAATATGAGCACAAGGTATTATTT	57	132–174	LC085276
Isu16408	(TC)18	[tail C] AGATTACTGCTTCGTTCGACCTTA	gtTTGGTGCTATAATTAGGATTTGGC	57	285–307	LC085277
Isu16655	(CT)16	[tail C] GAAAGGTAGGTCCATAACTCCACA	gtTTGAGGATACAATGCTTTCACTTG	57	270–290	LC085278
Isu16805	(GA)26	[tail B] CGCTCTTAAACAGAATATGGAAGG	gtttGATTGTCAATTCCACGGAGAAC	57	83–115	LC085279
Isu17435	(AG)20	[tail B] TAAATACAAAGATGATGTGCCAGC	gttTGTACATGTAGTTCCCAGGCAAT	57	82–114	LC085280
Isu17619	(AG)13	[tail A] CAATTCCCTTGTGAAGAATTATCG	gtttGTTTACAGTACTGCACTGACGCAT	57	317–329	LC085281

*T*a = annealing temperature

^a^Tails of the forward primers are indicted as follows: [Tail A] = 5′-GCCTCCCTCGCGCCA-3′; [Tail B] = 5′-GCCTTGCCAGCCCGC-3′; [Tail C] = 5′-CAGGACCAGGCTACCGTG-3′; and [Tail D] = 5′-CGGAGAGCCGAGAGGTG-3′

^b^Reverse primer sequences contained the PIG-tail sequence [2]. Tail sequences are shown in lower case letters

PCR amplification was performed using the QIAGEN Multiplex PCR Kit. Multiplex PCRs were performed for each of the four primer pair sets using the following thermal cycle conditions: initial denaturation for 15 min at 95° C, 35 cycles of denaturation for 30 s at 95° C, annealing for 1.5 min at 57° C, extension for 1 min at 72° C, and final extension for 30 min at 60° C. The PCR products were separated by capillary electrophoresis on an ABI3130 Genetic Analyzer (Life Technologies, Waltham, MA, USA) with the GeneScan 600 LIZ Size Standard (Life Technologies). The fragments were sized using GeneMapper 4.0 (Life Technologies).

We finally tested two populations from Chichijima and Hahajima Islands in the central part of the Ogasawara Islands to evaluate the allelic polymorphisms: 24 individuals from Asahiyama (27°05′40.7″N 142°12′35.6″E) on Chichijima Island and 20 individuals from Omotohama (26°37′28.9″N 142°10′41.7″E) on Hahajima Island. Voucher specimens of the representative individuals were deposited in the Makino Herbarium (MAK) of the Tokyo Metropolitan University, Japan (Asahiyama: no. MAK436933; Omotohama: no. MAK436934). The number of alleles per locus (*N*_A_), observed heterozygosity (*H*_O_), expected heterozygosity (*H*_E_), and fixation index (*F*_IS_) were calculated to characterize each locus using GenAlEx 6.501 [13]. The Hardy–Weinberg equilibrium (HWE) at each locus of each population and linkage disequilibrium (LD) between each locus pair in each population were tested with Genepop 4.0 [16]. In addition, the null allele frequencies (*F*_Null_) were estimated with CERVUS 3.07 [10]. To examine genetic differentiation between the two populations, Weir and Cockerham’s [19] estimate of pairwise *F*_ST_ was calculated using FSTAT 2.9.3.2 [6]. The deviation of each pairwise *F*_ST_ from zero was tested based on 1000 randomizations. Genetic structure was also evaluated by a Bayesian clustering method implemented in STRUCTURE 2.3.4 [4, 5, 15]. Markov chain Monte Carlo methods consisted of 100,000 burn-in steps and followed by 100,000 iterations. Ten replicate runs were performed at each *K* value from one to five under an admixture model with correlated allele frequencies. The log-likelihood probability at each run and the rate of change in the log-likelihoods between adjacent *K* values, *ΔK* [3], were calculated and compared across a range of *K* values to determine the best fit for the data.

### Results and discussion

Of the 48 tested microsatellite markers, 32 primer pairs were polymorphic among 44 individuals (Table 1). *N*_A_ ranged from three to 22 alleles in the Chichijima population and from one to nine alleles in the Hahajima population (Table 2). *H*_E_ ranged from 0.156 to 0.940 in the Chichijima population and from 0.368 to 0.845 in the Hahajima population (Table 2). Locus Isu07063 in the Hahajima population was monomorphic; only one allele was found in six samples, and the remaining 14 samples were not successfully amplified, suggesting the existence of null alleles. In addition, *F*_Null_ was high (Table 2). The Isu00524 locus in both populations deviated significantly from HWE. Significant deviations from HWE in the Chichijima or Hahajima populations were detected at several loci (Table 2; Isu04069, Isu07049, Isu10193, Isu12265, Isu15054, and Isu16805). These loci possibly involved null alleles, because null alleles are a common cause of apparent deviations from HWE [14]. Actually, *F*_Null_ values were high in most of these loci (Table 2). However, these HWE deviations may have been caused by inbreeding, which can often occur in small populations. In either case, these loci should be used cautiously in further analyses. No significant LD was observed between the markers in the two populations.

**Table 2:** Genetic diversity of the 32 microsatellite markers in the two *Distylium lepidotum* populations

Locus	Chichijima Island					Hahajima Island					*F*_Null_
	*N*	*N*_A_	*H*_O_	*H*_E_	*F*_IS_^a^	*N*	*N*_A_	*H*_O_	*H*_E_	*F*_IS_^a^	
Isu00524	22	5	0.182	0.381	0.523*	20	5	0.450	0.650	0.308*	0.265
Isu01062	24	19	0.917	0.925	0.009	20	9	0.850	0.829	−0.026	0.018
Isu01853	24	12	0.875	0.891	0.018	20	8	0.750	0.836	0.103	0.038
Isu03838	24	8	0.625	0.800	0.219	20	6	0.700	0.749	0.065	0.116
Isu04069	24	9	0.375	0.793	0.527***	20	6	0.550	0.551	0.002	0.249
Isu04385	24	14	0.917	0.884	−0.037	20	7	0.950	0.788	−0.206	−0.032
Isu04423	24	16	0.750	0.844	0.111	20	8	0.850	0.826	−0.029	0.045
Isu04472	24	18	0.958	0.913	−0.049	20	6	0.600	0.613	0.020	0.026
Isu04870	24	4	0.833	0.702	−0.187	20	4	0.700	0.638	−0.098	−0.057
Isu04950	24	7	0.625	0.661	0.055	20	9	0.950	0.830	−0.145	0.050
Isu04954	24	7	0.583	0.582	−0.003	20	5	0.750	0.678	−0.107	0.032
Isu05730	24	8	0.833	0.816	−0.021	20	6	0.800	0.771	−0.037	−0.004
Isu06843	24	14	0.875	0.886	0.013	20	8	0.900	0.805	−0.118	−0.004
Isu07049	24	15	0.833	0.917	0.091	20	8	0.550	0.746	0.263*	0.109
Isu07063	17	9	0.235	0.843	0.721***	6	1	–	–	–	0.659
Isu09807	24	13	0.750	0.788	0.048	20	5	0.850	0.726	−0.170	−0.001
Isu09853	24	7	0.625	0.787	0.206	20	8	0.700	0.756	0.074	0.112
Isu10193	24	9	0.750	0.848	0.116	20	7	0.400	0.770	0.481**	0.174
Isu11459	24	8	0.625	0.500	−0.250	20	4	0.400	0.368	−0.088	−0.104
Isu12115	24	3	0.333	0.588	0.433	20	3	0.700	0.609	−0.150	0.141
Isu12238	24	12	0.958	0.858	−0.117	20	7	0.600	0.693	0.134	0.019
Isu12265	24	13	0.583	0.845	0.310**	20	7	0.800	0.800	0.000	0.126
Isu12586	24	14	0.875	0.862	−0.015	20	9	0.650	0.769	0.154	0.051
Isu13849	24	9	0.875	0.780	−0.122	20	4	0.500	0.524	0.045	−0.014
Isu13965	24	12	0.875	0.885	0.012	20	6	0.800	0.769	−0.041	0.010
Isu15054	24	22	0.833	0.940	0.114*	20	8	0.800	0.845	0.053	0.060
Isu16246	24	12	0.667	0.840	0.207	20	9	0.800	0.836	0.043	0.087
Isu16408	24	9	0.917	0.842	−0.089	20	7	0.600	0.578	−0.039	0.046
Isu16655	24	10	0.667	0.789	0.155	20	7	0.750	0.800	0.063	0.073
Isu16805	24	11	0.500	0.857	0.416*	20	8	0.500	0.701	0.287	0.284
Isu17435	24	12	0.833	0.838	0.005	20	6	0.800	0.703	−0.145	0.014
Isu17619	24	3	0.167	0.156	−0.067	20	3	0.600	0.496	−0.209	−0.047
Average	–	10.8	0.695	0.776	0.105	–	6.4	0.675	0.689	0.016	–

*N* = number of genotyped individuals; *N*_A_ = number of alleles per locus; *H*_O_ = observed heterozygosity; *H*_E_ = expected heterozygosity; *F*_IS_ = fixation index; *F*_Null_ = null allele frequency

^a^ Asterisks indicate significant deviation from Hardy–Weinberg equilibrium after Bonferroni correction (^*^ *P* < 0.05, ^**^ *P* < 0.01, ^***^ *P* < 0.001)

**Fig. 1:**
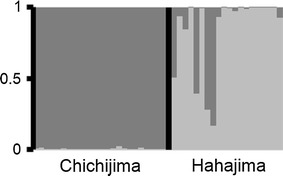
Results of Bayesian clustering, STRUCTURE, at *K* = 2 of the two *Distylium lepidotum* populations. *Vertical columns* represent individual plants, and the heights of *bars* of each *color* are proportional to the posterior means of estimated admixture proportions. For population localities, see Table 1

Of all the 397 alleles that were detected, the 193 alleles which were detected in the Chichijima population were not found in the Hahajima population. On the other hand, the 53 alleles which were detected in the Hahajima population were not found in the Chichijima population. In addition, the two populations were significantly differentiated (*F*_ST_ = 0.0971). The Bayesian clustering analysis represented the highest *ΔK* value at *K* = 2 (*ΔK* = 121.4; Appendix). The Chichijima population was almost entirely composed of the cluster I (dark gray); the Hahajima population generally comprised the cluster II (light gray) (Fig. 1). However, because admixture was observed in some individuals of the Hahajima population, the infrequent gene flow between islands might occur. These data indicated that these markers can be used to analyze population genetic structure in the future.

### Conclusions

These 32 novel microsatellite markers will be valuable for elucidating the genetic diversity and structure of *D. lepidotum*, since they have enough polymorphisms and they can clearly distinguish the two populations. The genetic data would be useful to investigate the genetic diversity and structure of *D. lepidotum* which is necessary for a food source of the endangered locust species on the Ogasawara Islands.

## Figures and Tables

**Fig. 2:**
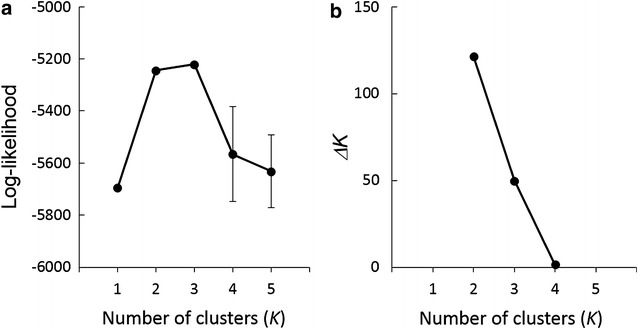
Results of Bayesian clustering, STRUCTURE, of the two *Distylium lepidotum* populations. **a** Changes in the log-likelihood, **b** *ΔK* as the number of clusters (*K* ranging from one to five)

## Appendix

See Fig. 2.

## Abbreviations

*F*_IS_: fixation index
*F*_Null_: null allele frequency
*H*_E_: expected heterozygosity
*H*_O_: observed heterozygosity
HWE: Hardy–Weinberg equilibrium
LD: linkage disequilibrium
*N*_A_: number of alleles per locus
NGS: next-generation sequencing
PCR: polymerase chain reaction

## References

[1] Blacket MJ, Robin C, Good RT, Lee SF, Miller AD (2012). Universal primers for fluorescent labelling of PCR fragments-an efficient and cost-effective approach to genotyping by fluorescence. Mol Ecol Res.

[2] Brownstein MJ, Carpten JD, Smith JR (1996). Modulation of non-templated nucleotide addition by taq DNA polymerase: primer modifications that facilitate genotyping. Biotechniques.

[3] Evanno G, Regnaut S, Goudet J (2005). Detecting the number of clusters of individuals using the software STRUCTURE: a simulation study. Mol Ecol.

[4] Falush D, Stephens M, Pritchard JK (2003). Inference of population structure using multilocus genotype data: linked loci and correlated allele frequencies. Genetics.

[5] Falush D, Stephens M, Pritchard JK (2007). Inference of population structure using multilocus genotype data: dominant markers and null alleles. Mol Ecol Notes.

[6] Goudet J. Fstat v2.9.3.2. Universitity of Lausanne, Lausanne. 2002. http://www2.unil.ch/popgen/softwares/fstat.htm. Accessed 12 Apr 2016.

[7] Hannon G. FASTX-Toolkit v.0.0.14. Cold Spring Harbor Laboratory, Long Island. 2009. http://hannonlab.cshl.edu/fastx_toolkit/. Accessed 12 Apr 2016.

[8] Ishikawa H (2011). Occurrence of a New Grasshopper, *Boninoxya anijimensis* gen. et sp. nov. (Orthoptera, Acrididae, Oxyinae) in the Ogasawara Islands, Japan. Jpn J Syst Entomol.

[9] Ishikawa H. *Boninoxya anijimensis*, In: Natural Environment Division, Bureau Of Environment, Tokyo Metropolitan Government, editors. Red Data Book Tokyo: Islands version. Natural Environment Division, Bureau of Environment, Tokyo Metropolitan Goverment, Tokyo; 2014. p. 400. **(in Japanese)**.

[10] Kalinowski ST, Taper ML, Marshall TC (2007). Revising how the computer program CERVUS accommodates genotyping error increases success in paternity assignment. Mol Ecol.

[11] Meglecz E, Costedoat C, Dubut V, Gilles A, Malausa T, Pech N, Martin J-F (2010). QDD: a user-friendly program to select microsatellite markers and design primers from large sequencing projects. Bioinformatics.

[12] Morgan M, Anders S, Lawrence M, Aboyoun P, Pages H, Gentleman R (2009). ShortRead: a bioconductor package for input, quality assessment and exploration of high-throughput sequence data. Bioinformatics.

[13] Peakall R, Smouse PE (2006). GENALEX 6: genetic analysis in excel. Population genetic software for teaching and research. Mol Ecol Notes.

[14] Pemberton JM, Slate J, Bancroft DR, Barrett JA (1995). Nonamplifying alleles at microsatellite loci—a caution for parentage and population studies. Mol Ecol.

[15] Pritchard J, Stephens M, Donnelly P (2000). Inference of population structure using multilocus genotype data. Genetics.

[16] Rousset F (2008). GENEPOP‘007: a complete re-implementation of the GENEPOP software for Windows and Linux. Mol Ecol Res.

[17] Rozen S, Skaletsky HJ, Misener S, Krawetz SA (2000). Primer3 on the WWW for general users and for biologist programmers. Bioinformatics methods and protocols.

[18] Shimizu Y, Tabata H (1991). Forest structures, composition, and distribution on a Pacific island, with reference to ecological release and speciation. Pac Sci.

[19] Weir BS, Cockerham CC (1984). Estimating *F*-statistics for the analysis of population-structure. Evolution.

[20] Zalapa JE, Cuevas H, Zhu H, Steffan S, Senalik D, Zeldin E, McCown B, Harbut R, Simon P (2012). Using next-generation sequencing approaches to isolate simple sequence repeat (SSR) loci in the plant sciences. Am J Bot.

[21] Zhang J, Kobert K, Flouri T, Stamatakis A (2014). PEAR: a fast and accurate Illumina Paired-End reAd mergeR. Bioinformatics.

